# Soft tissue can absorb surprising amounts of energy during knee exoskeleton use

**DOI:** 10.1098/rsif.2024.0539

**Published:** 2024-12-04

**Authors:** W. Sebastian Barrutia, Ada Yumiceva, Mai-Ly Thompson, Daniel P. Ferris

**Affiliations:** ^1^ Crayton Pruitt Family Department of Biomedical Engineering, University of Florida, Gainesville, FL, USA

**Keywords:** exoskeleton, mechanical phantom, human–exoskeleton interface, energetics, soft-tissue deformation, synthetic limb

## Abstract

Soft tissue at the human–exoskeleton interface can deform under load to absorb, return and dissipate the mechanical energy generated by the exoskeleton. These soft tissue effects are often not accounted for and may mislead researchers on the actual joint assistance an exoskeleton provides. We assessed the effects of soft tissue by quantifying the performance and energy distribution of a knee exoskeleton under different assistance strategies using a synthetic lower limb phantom. The phantom emulated knee kinematics and soft tissue deformation at the exoskeleton interface. We loaded the exoskeleton on the phantom under six different spring stiffness conditions. Motion capture marker and load cell data from the phantom–exoskeleton assembly allowed us to estimate the moments, stiffness and energy contributions of the exoskeleton and physical interface. We found that soft tissue caused interface power to increase and exoskeleton power to decrease with increasing spring stiffness. Despite similar joint kinematics, our findings show that increasing exoskeleton assistance did not notably change power transfer to the targeted joint, as soft tissue compressed under high forces. Our methodology improves exoskeleton design process by estimating energy distribution and transfer for exoskeletons while accounting for the effects of soft tissue deformation before human testing.

## 1. Introduction

Assistive forces travelling from the exoskeleton to the knee go through the thigh and shank, body segments containing high volumes of soft tissue (i.e. muscle, fat, skin) with viscoelastic properties that deform under load [1,2]. As a result, soft tissue can absorb, return and dissipate exoskeleton mechanical energy, decreasing its efficacy in assisting the joint. This mismatch between exoskeleton power output and the assistive power transmitted to the joint may mislead exoskeleton researchers on the actual benefits of their assistive devices, potentially hindering design engineering development. Researchers and engineers must address these limitations to increase the chances of producing a successful device.

Testing human users of robotic exoskeletons during prototype development can be time and effort intensive, especially when targeting populations such as patients or children for exoskeleton use. Alternatively, researchers can use mechanical synthetic limb phantoms to improve the design and engineering process for robotic exoskeletons. Synthetic phantom limbs can allow engineers to assess device performance, safety and reliability prior to human subject testing [3–7]. The advantages of characterizing the performance of an exoskeleton on a phantom include the lack of subject heterogeneity and safety concerns regarding testing on a vulnerable population. Furthermore, the absence of biological moments makes it relatively simple to calculate the assistance provided by the device and experienced by the joint. While helpful, mechanical phantoms are often treated as rigid bodies that do not deform under load, leading to a mismatch between the estimated exoskeleton assistance and the actual assistance experienced by its user [8,9]. One alternative is to use a mechanical phantom that replicates soft tissue deformation. We have previously developed a mechanical phantom capable of emulating sagittal-plane knee kinematics and soft-tissue deformation at the exoskeleton interface [10]. Such a mechanical phantom would enable us to more accurately characterize the performance of a knee exoskeleton assisting children with crouch gait before human trials.

The objective of this study was to use a mechanical lower-limb synthetic phantom to measure the energy distribution and assistance profile of a pediatric knee exoskeleton. The mechanical phantom used a robotic platform to replicate the knee kinematics of crouch gait and had ballistic gel simulating soft tissue deformation at the human–exoskeleton interface. We chose to replicate crouch gait knee kinematics, as future work in the laboratory will examine the effects of walking with the exoskeleton on children with crouch gait caused by cerebral palsy. We identified sites of mechanical energy loss within the exoskeleton and its human interface for a range of exoskeleton spring stiffnesses, providing researchers with insights on improving knee exoskeleton performance.

## 2. Methods

### 2.1. Passive exoskeleton design

**Figure 1.**
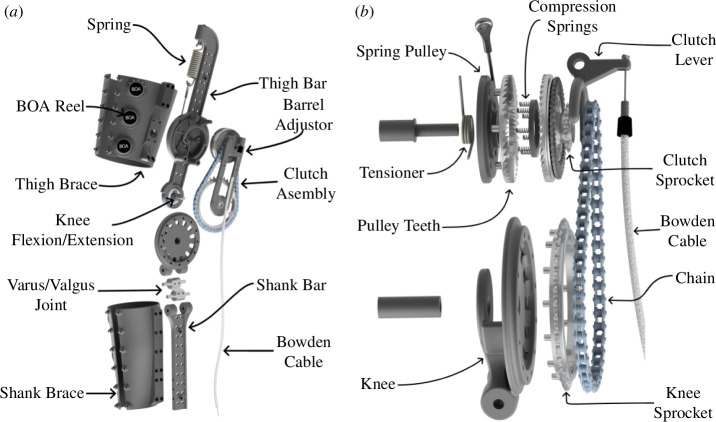
Exoskeleton assembly. (*a*) Render of the exoskeleton frame and straps. The device consisted of two bars parallel to the lower limb. (*b*) Exploded view of the exoskeleton clutch, which engaged the spring when the Bowden cable was pulled and disengaged it when the cable tension was released.

We designed the exoskeleton to emulate the typical behaviour of the human knee during the gait cycle. A healthy human knee exerts a knee extensor moment proportional to the change in knee flexion in the early and middle portions of stance phase during gait. Unlike stance, the swing phase presents a nonlinear relationship between the knee moment and angle [11]. Bio-inspired by the behaviour of the human knee, we developed an exoskeleton that used an extension spring to exert a knee extensor moment during stance (figure 1*a*). The device consisted of two bars parallel to the user’s thigh and shank, which were attached to the human leg using semi-compliant straps. The extension spring is attached to a spring pulley, which is connected to the knee joint via an interference clutch and a chain-sprocket transmission. With the clutch engaged, knee flexion elongated the spring and resulted in a knee extensor moment supporting the joint.

The exoskeleton engaged its spring during stance and disengaged it during the leg swing phase to allow unobstructed knee motion. A mechanical dog clutch comprising interlocking teeth connected the spring to the chain transmission (figure 1*b*). Compression springs between the set of teeth kept the spring disconnected from the transmission by default. A low-stiffness tensioner torsion spring attached to the spring pulley provided a pre-tension to the extension spring. A Bowden cable pulling on a clutch lever can push the interlocking teeth together to engage the clutch to connect the spring to the knee. This cable pulling can be accomplished during the stance phase mechanically (e.g. with the cable attached to a mechanical foot pedal) or electromechanically (e.g. a cable attached to a servo motor detecting foot strike via force sensors at the shoe).

**Figure 2.**
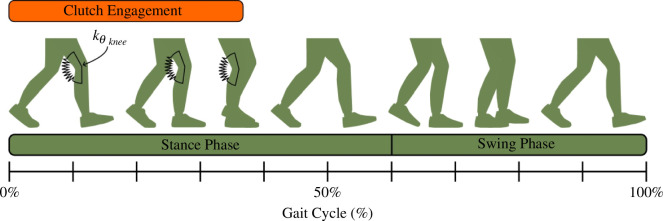
Exoskeleton activation across the gait cycle. The exoskeleton behaves like a torsion spring with a stiffness knee to support the knee during early to middle stance phase during weight acceptance. Clutch disengagement during terminal stance allows unconstrained knee motion for the rest of the gait cycle.


The resulting device provided a knee extensor moment during stance and no resistance during leg swing (figure 2). As shown in equation (2.1), the theoretical extensor moment M (Nm) was 0 during leg swing. During stance, the moment resulted from a proportionality constant $k_{\theta_{knee}}$ (Nm rad^−1^) and a change in knee flexion angle $\Delta \theta_{knee}\;(rad)$ relative to the angle at the time at which the clutch was engaged. As shown in equation (2.2), the proportionality constant $k_{\theta_{knee}}$ was a function of the extension spring stiffness $k_{l}$ (N m^−1^), tensioner spring stiffness $k_{\theta_{tensioner}}$ (Nm rad^−1^), spring pulley radius r (m) and transmission ratio ${\left(T/t\right)}^{2}$.

(2.1) $$M=\left\{ \begin{array}{l} k_{\theta_{knee}}\Delta \theta_{knee}\\ 0 \end{array}\right.\;\begin{array}{c} \left(during\;stance\right)\\ \left(during\;swing\right), \end{array}$$

(2.2) $$k_{\theta_{knee}}=\left(k_{l}-\frac{k_{\theta_{tensioner}}}{r^{2}}\right){\left(\frac{T}{t}\right)}^{2}r^{2}.$$

We designed the exoskeleton to provide as much comfort to its user [12]. A varus/valgus hinge below the knee joint reduced device misalignment in the frontal plane. Besides the additional degrees of freedom, the straps were semi-compliant to fit several leg sizes and shapes. L6 BOA® Fit System reels attached to each strap tightened them around its user’s lower limb. We constructed the device with lightweight materials to decrease its carrying weight. Most of the exoskeleton frame was made from 3D-printed nylon reinforced with continuous carbon fibre. A metal laser sintering printer manufactured small parts expected to withstand relatively high loads, such as the sprockets, varus/valgus joint and dog clutch teeth. Per limb, the final device weighed approximately 1.1 kg.

### 2.2. Phantom limb design

The synthetic phantom replicated the morphology and soft-tissue deformation of a human lower limb. We have previously described the construction and testing of the mechanical phantom, which we constructed from three-dimensional body shapes and bone models. The body model was downloaded from the HumanShape™ library and corresponded to a standing child model with a height and body mass index of 1.31 m and 17 kg m^−2^ [13]. We downloaded three-dimensional bone models of the femur, tibia and fibula from The Living Human Digital Library and fitted them inside the body shape model as we have described previously [10,14]. Using Autodesk Inventor Professional (Autodesk, San Fransisco, CA), we segmented the lower limb of the body/bone model into thigh and shank segments and replaced the ankle, knee and hip joints with ball-bearing hinges. The bearings at each of the phantom joints had the same axis of rotation, which allowed sagittal plane motion with minimal friction [15]. We 3D-printed the femur, tibia and fibula bones with nylon and reinforced them with continuous carbon fibre using a Markforged X7 (Markforged Inc., Waltham, MA). We then poured a mixture of 15% (w/v) ballistic gelatin into thigh and shank moulds constructed from the three-dimensional body model. Ballistic gelatin has similar mechanical properties to human soft tissue, and we previously found that 15% (w/v) gelatin had a stiffness within the range of human lower-limb muscles [2,10,16,17].

### 2.3. Experimental set-up

**Figure 3.**
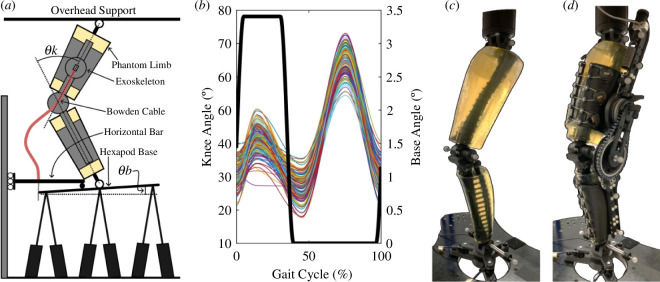
Exoskeleton–phantom set-up. (*a*) Drawing of the exoskeleton and phantom assembly. Moving the phantom ankle vertically while keeping the hip fixed allowed the knee to flex and extend. Rotation of the hexapod base pulled on the Bowden cable and engaged the exoskeleton clutch. (*b*) Plots of the phantom knee (coloured lines) and hexapod base (thick black line) angles emulated by the hexapod. Each cycle lasted 2 s. (*c*) Phantom limb assembly with passive motion capture markers and ankle load cells attached. (*d*) Phantom/exoskeleton assembly with additional exoskeleton motion capture markers and spring load cell.

The synthetic phantom leg emulated the kinematics of crouch gait. As shown in figure 3*a*, we fixed the phantom hip to a stationary overhead support while vertically attaching the ankle to a NOTUS hexapod base (Symetrie, Nîmes, France). As discussed in Barrutia *et al*., the upward and downward motion of the hexapod base resulted in phantom knee flexion and extension, respectively [10]. We programmed the phantom knee to follow a crouch gait trajectory based on the subjects described in Lerner *et al*. [18]. Biomechanical data of subjects were collected at Gillette Children’s Specialty Healthcare and corresponded to individuals with spastic diplegic cerebral palsy with the following inclusion criteria: (i) at least one clean individual limb force plate strike, (ii) minimum knee flexion during stance between 15 and 30°, and (iii) less than 30° of tibial or femoral torsion [19,20]. The parents and guardians provided informed consent prior to data collection. Using MATLAB (MathWorks, r2023a), we extracted the mean knee flexion peaks and valleys along their stride and used a shape-preserving piecewise cubic interpolation function to reconstruct a representative crouch gait trajectory. To ensure that the exoskeleton was robust among a range of kinematic trajectories, we added stochastic randomness to each knee flexion peak and valley from a normal distribution centred about 0 with a 4° standard deviation. Tabard-Fougère *et al*. previously found a maximum intrinsic variability of 4° of root mean square deviation in the knee flexion of children with cerebral palsy during gait [21]. We computed 150 random knee trajectories over a 100 Hz sampling frequency and a 2 s stride time and exported them into SYM_Motion software (Symetrie, Nîmes, France) to execute the respective hexapod motions. These 150 variable trajectories were kept constant among all the exoskeleton testing conditions. We chose a 2 s stride time due to speed limitations of the hexapod hardware. While we introduced knee angle variability, the knee flexion peak and valley timings and total stride time were kept constant throughout the experimental procedure. Figure 3*b* presents the random crouch gait trajectories emulated using the phantom lower limb.

We attached the exoskeleton to the phantom limb and engaged its clutch during stance. To pull on the Bowden cable and engage the clutch, we used a bar that remained horizontal and moved vertically with the hexapod base. We attached the outer tube of the Bowden cable to the horizontal bar and the inner wire to the hexapod base so that tilting the hexapod base resulted in cable pulling and clutch engagement (figure 3*a*). Using SYM_Motion, we programmed the hexapod base to tilt 3.4° during early and middle stance (0–35% of the gait cycle) using a smooth step function. As a result, the hexapod moved vertically to flex and extend the phantom knee and tilted its base to engage and disengage the exoskeleton clutch (figure 3*b*).

We used the phantom limb to assess exoskeleton performance in various spring stiffness conditions. For the same variable knee trajectories, we recorded force and motion data of the phantom/exoskeleton assembly under six different springs rated at 3.4, 6.1, 9.2, 13.6, 18.0 and 21.7 kN m^−1^. To reduce bias, we randomized the order of spring condition tested: 9.2, 21.7, 13.6, 6.1, 3.4 and 18.0 kN m^−1^. The phantom and exoskeleton contained six passive motion capture markers each. The ankle, knee and hip joints of the phantom had a marker at their medial and lateral sides. The exoskeleton had two markers at its thigh and shank bars and a single marker at each end of its spring (figure 3*d*). Four Optitrack cameras recorded each marker position at 100 Hz. We instrumented the phantom/exoskeleton assembly with load cells to estimate forces within the system. Three REB7 compression/tension load cells (Loadstar Sensors, Freemont, CA) at the phantom ankle recorded vertical external forces against the hexapod base. An LC703 load cell (Omega, Norwalk, CT) attached in series with the spring recorded spring force during each trial. All load cell readings were recorded at 1000 Hz. Electronic supplementary material, video S1 shows the hexapod moving the phantom/exoskeleton assembly for the 3.4 kN m^−1^ spring.

### 2.4. Data processing

We used MATLAB to process and analyse all recorded data to estimate the kinematics and kinetics of the phantom/exoskeleton system. Due to the hexapod speed ramp-up and slow-down, the initial and final strides were excluded, resulting in 140 strides for analysis. Motion capture and load cell data were filtered with a fourth-order zero-phase Butterworth filter with a 6 Hz cut-off frequency. We calculated spring force and elongation from the spring load cell and three-dimensional markers positions. We then excluded strides with less than 5 N of spring force to assess exoskeleton performance during strides with spring elongation, resulting in 126 out of the 140 strides being selected for further analysis. We spline interpolated all marker position data to 1000 Hz to match the load cell acquisition frequency. We projected the exoskeleton (thigh and shank excluding spring) and phantom joint markers into the sagittal plane to calculate the phantom/exoskeleton flexion angles and linear/angular accelerations over time. To estimate the phantom and exoskeleton knee range of motion during the stance phase, we calculated the stance knee flexion as the difference between the peak and initial stride angles during midstance. A custom inverse dynamics model discussed in Barrutia *et al*. used the ankle load cell forces, phantom body segment parameters and inertial forces to estimate the moment experienced by the phantom knee at each time point [10].

We further analysed the exoskeleton and phantom kinematics and kinetic data to estimate the stiffness experienced by each. The inverse dynamics model estimated the net knee moments experienced by the phantom knee. Assuming negligible frictional forces, the phantom knee was a passive hinge with no biological muscles or tendons, and we assumed that its moments came entirely from the exoskeleton. We estimated two assistance profile types by plotting the same knee moments over the phantom and exoskeleton knee angles across all spring stiffness conditions. A linear regression model of the resulting moment-angle plots during the spring-loading portion of the stance phase estimated the stiffness of the exoskeleton and phantom limb while the exoskeleton was engaged. Unlike the exoskeleton stiffness, the phantom stiffness incorporated the effects of soft-tissue deformation.

**Figure 4.**
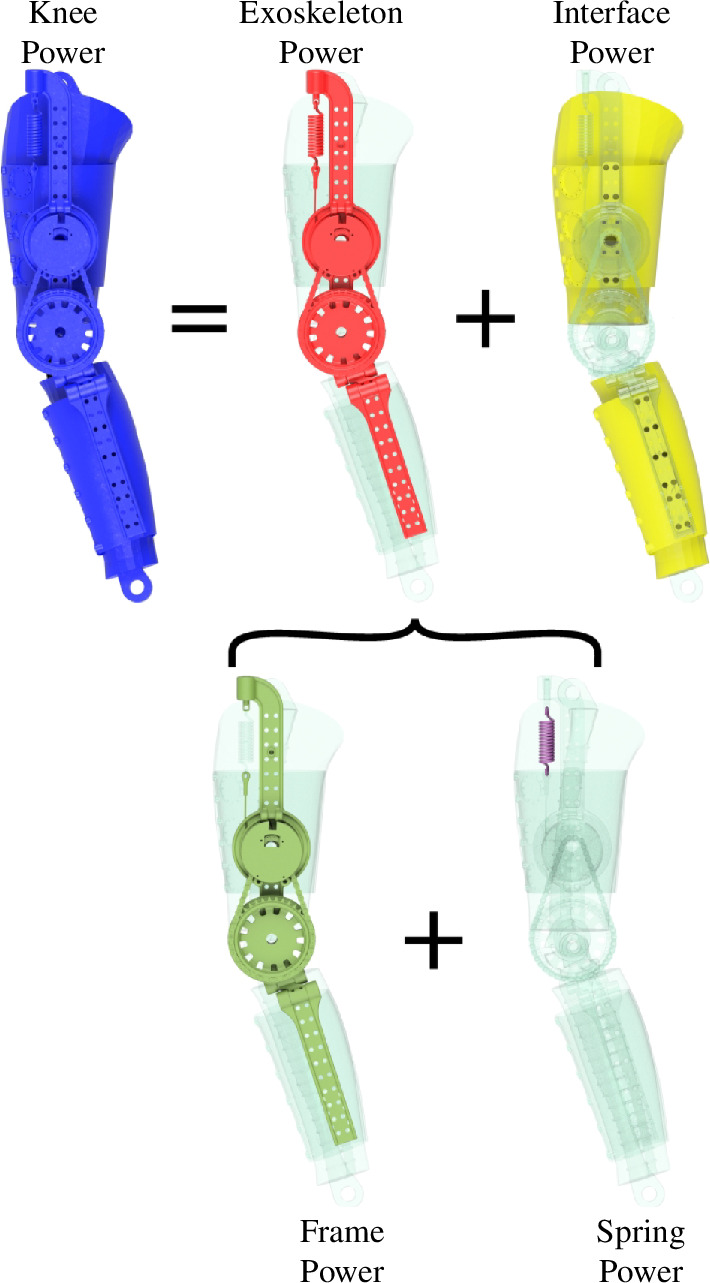
Energy distribution conceptualization. The knee power (blue) represents the total energy within the phantom/exoskeleton assembly. The knee power contains exoskeleton (red) and physical interface (yellow) power components. Similarly, the exoskeleton comprises the frame (green) and the spring (purple). We estimated the energy contained within each component to assess exoskeleton performance.


We estimated the energy distribution within the phantom/exoskeleton system to identify areas of energy storage, return and loss. We partitioned the assembly into five components: knee, exoskeleton, interface, frame and spring (figure 4). The knee component contained the entire phantom/exoskeleton assembly and represented the power provided to the phantom knee and hypothetical human knee. The knee component contained exoskeleton and interface subcomponents. The exoskeleton component comprised the exoskeleton but excluded the thigh and shank straps. The interface component contained the phantom ballistic gel alongside the exoskeleton straps. We included the exoskeleton straps in the interface component since the exoskeleton power was estimated from the motion of markers attached to the exoskeleton thigh and shank bars. Exoskeleton strap deformation would not be captured by these markers. We excluded the 3D-printed bones from the interface power component as these were built with continuous carbon fibre, and we expected them to have negligible deformation relative to the ballistic gel and exoskeleton straps during the experimental trials. We estimated the knee power $P_{knee}\;\left(W\right)$ and exoskeleton power $P_{exoskeleton}\;\left(W\right)$ by multiplying the knee moment M (Nm) with the phantom ${\overset{˙}{\theta }}_{phantom}$ (rad s^−1^) and exoskeleton ${\overset{˙}{\theta }}_{exoskeleton}$ (rad s^−1^) knee angular speeds, respectively. The interface power $P_{interface}$ was calculated as the difference between the knee and exoskeleton power, equation (2.3),

(2.3) $$\begin{array}{l} P_{knee}=P_{exoskeleton}+P_{interface}\\ M{\overset{˙}{\theta }}_{phantom}=M{\overset{˙}{\theta }}_{exoskeleton}+P_{interface}. \end{array}$$

Similarly, we divided the exoskeleton into frame and spring components. The frame component comprised the exoskeleton bars, dog clutch and chain transmission, excluding the exoskeleton straps and spring. We estimated the spring power $P_{spring}\;\left(W\right)$ by multiplying the spring force $F_{spring}\;\left(N\right)$ with the spring deflection rate ${\overset{˙}{l}}_{spring}$ (m s^−1^).The frame power $P_{frame}\;\left(W\right)$ was estimated from the difference between the exoskeleton and spring powers, equation (2.4),


(2.4) $$\begin{array}{l} P_{exoskeleton}=P_{frame}+P_{spring}\\ M{\overset{˙}{\theta }}_{exoskeleton}=P_{frame}+F_{spring}{\overset{˙}{l}}_{spring}. \end{array}$$

We estimated the negative, positive and net work of each phantom/exoskeleton component by integrating their respective powers over time.

### 2.5. Statistical analysis

Using R 4.3.2, we performed a Krustal–Wallis test on the stance knee flexion angle, peak stance moments, phantom/exoskeleton stiffness and net/negative/positive work values. We chose a non-parametric test due to the non-normal distribution and variance non-homogeneity of the data. Post hoc Dunn’s tests adjusted with Bonferroni correction tested for multiple comparisons across the spring stiffness conditions. Electronic supplementary material, tables S1–S8 summarize the Krustal–Wallis and post hoc Dunn’s test results.

## 3. Results

### 3.1. Kinematics

**Figure 5.**
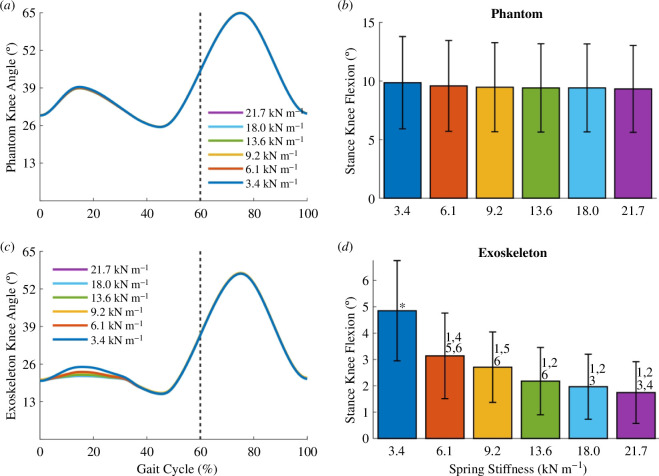
Exoskeleton–phantom kinematics. (*a*) Plots of the mean phantom knee angle over the gait cycle for all spring stiffness conditions. The dotted vertical line represents the transition from stance to swing phase. (*b*) Bar plots of the mean stance knee flexion of the phantom for all spring conditions. We define the stance knee flexion as the difference between the peak knee flexion in midstance and the initial knee flexion angle of each stride. (*c*) For all spring conditions, plots of the mean exoskeleton knee angle over the gait cycle. (*d*) Bar plots of the mean exoskeleton stance knee flexion for all spring conditions. Error bars denote standard deviations. (1), (2), (3), (4), (5), (6) = significantly different from springs 3.4, 6.1, 9.2, 13.6,18.0, 21.7 kN m^−1^, respectively. (*) = significantly different from all other groups. The *p*-values for the Krustal–Wallis and all post hoc tests are specified in electronic supplementary material, table S1.

The change in phantom knee kinematics was negligible across the more than sixfold change in spring stiffness conditions. Figure 5*a* shows the mean phantom limb knee angles over the gait cycle for each spring stiffness condition. Figure 5*b* shows the mean stance knee flexion, defined as the difference between the peak knee flexion angle during midstance and the initial knee flexion angle of the stride. The differences in the phantom stance knee flexion across conditions were small and not statistically significant. For example, the medians of the phantom stance knee flexion of the 3.4 and 21.7 kN m^−1^ springs only differed by 0.6°.

The exoskeleton knee angle during the stance phase differed across the spring conditions. During exoskeleton engagement at stance, the mean exoskeleton knee angle decreased with increasing spring stiffness (figure 5*c*). Figure 5*d* shows that the stance knee flexion of the exoskeleton was significantly different across the spring stiffness extremes. As a comparison across extremes, the median exoskeleton stance knee flexion of the 3.4 and 21.7 kN m^−1^ springs were significantly different from each other (4.9 and 1.6°, respectively; Dunn’s post hoc test, *p* < 0.0001, *n* = 126; figure 5*d*).

The range of motion of the exoskeleton knee was lower than that of the phantom limb (figure 5*b* and *d*). For the 3.4 kN m^−1^ spring, the median phantom and exoskeleton stance knee flexion angles were 10.1 and 4.9° respectively, approximately a twofold decrease. The difference between exoskeleton flexion and phantom flexion occurred because of soft tissue compression of the phantom. This difference between phantom and exoskeleton knee angle increased with increasing spring stiffness. For the 21.7 kN m^−1^ spring, the median phantom and exoskeleton stance knee flexion angles were 9.5 and 1.6°, approximately a sixfold drop in knee flexion.

### 3.2. Kinetics

**Figure 6.**
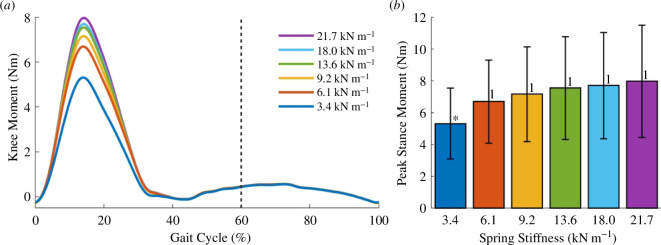
Kinetics. (*a*) For every spring stiffness, plots of the mean exoskeleton moments over the entire gait cycle. The exoskeleton provided knee extensor moments during early stance up to midstance while providing little to no resistance during leg swing. The dotted vertical line represents the transition from stance to swing phase. (*b*) Bar plots show the mean peak stance knee moments of all spring conditions. Error bars denote standard deviations. (*) = significantly different from all other groups. (1) = significantly different from 3.4 kN m^−1^ spring. The *p*-values of all statistical tests are specified in electronic supplementary material, table S2.

The exoskeleton produced extensor knee moments during the stance phase of gait while giving little to no resistance to the knee joint during the swing phase. Figure 6*a* shows the mean knee moments over the gait cycle across all spring conditions. The extensor moments increased and peaked during midstance while the exoskeleton was engaged. Only the peak knee moment of the 3.4 kN m^−1^ spring was significantly different from the remaining conditions (figure 6*b*). This peak moment did not increase proportionally to the change in spring stiffness. At the extremes, the median peak moments of the 3.4 and 21.7 kN m^−1^ springs were 5.5 and 8.0 Nm, respectively. In this scenario, a more than sixfold increase in spring stiffness only increased the exoskeleton of the knee moment by approximately 50%.

### 3.3. Assistance profile

We estimated the assistance profile for the phantom limb and exoskeleton knee joints by plotting the knee moments over the phantom and exoskeleton knee angles. From these plots, we calculated the phantom and exoskeleton knee stiffnesses, which we define as the best linear fit of the moment-angle stance plot during exoskeleton loading. Unlike the exoskeleton stiffness, the phantom stiffness incorporated the effects of soft-tissue deformation.

**Figure 7.**
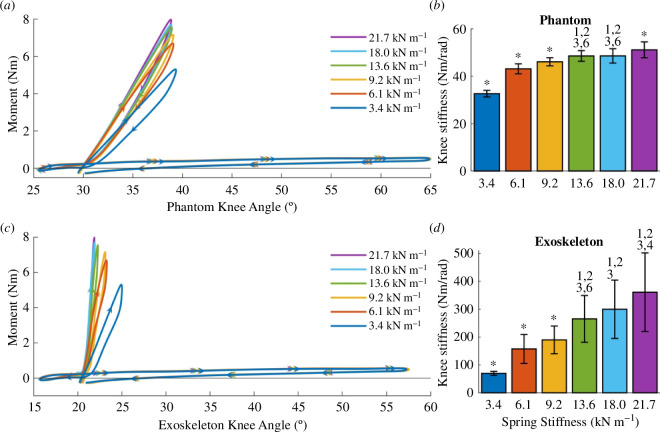
Assistance profiles. (*a*) For each spring stiffness, phantom work loops show the mean moments generated by the exoskeleton over the phantom knee joint. The steep work loops correspond to the stance phase of gait. (*b*) Mean phantom work loop stiffness (slope) during stance phase loading. (*c*) Plots of the mean exoskeleton work loops showing the moments over the exoskeleton knee angle. (*d*) Mean stiffness of the exoskeleton work loops during exoskeleton loading. Error bars denote the standard deviations. (*) = significantly different from all other groups. (1), (2), (3), (4), (5), (6) = significantly different from springs 3.4, 6.1, 9.2, 13.6, 18.0, 21.7 kN m^−1^, respectively. The *p*-values for all statistical tests are specified in electronic supplementary material, table S3.

The phantom limb experienced a knee extensor moment proportional to the change in knee flexion during the stance phase. Figure 7*a* shows the mean knee moment over the phantom knee angle across all spring conditions. A single stride contained two work-loops, with the steeper one corresponding to the stance phase and the flat one representing the swing phase. The swing phase work-loops showed that the phantom experienced little resistance during swing. From the assistance profile, we calculated the stance phantom joint stiffness, which increased with increasing spring stiffness (figure 7*b*). However, this increase in joint stiffness was not proportional to the change in spring stiffness. For the 3.4 and 21.7 kN m^−1^ springs, the median knee stiffness values were significantly different at 32.7 and 51.6 Nm rad^−1^, respectively (Dunn’s post hoc test, *p* < 0.0001, *n* = 126; figure 7*b*). While the spring stiffness increased by more than six times, the phantom knee stiffness during stance only increased by approximately 60%.

The exoskeleton knee stiffness increased with increasing spring stiffness. Figure 7*c* shows the mean knee moment over the exoskeleton knee angle across the spring conditions. Like the phantom knee, the exoskeleton knee underwent two work-loops for every stride, with the steep one corresponding to the stance phase. During stance, the exoskeleton knee stiffness increased with increasing spring stiffness (figure 7*d*). Unlike the phantom knee joint stiffness, which plateaued with increasing spring stiffness, the exoskeleton joint stiffness increased proportionally with spring stiffness. For the 3.4 and 21.7 kN m^−1^ springs, the median exoskeleton stiffness values were significantly different at 69.7 and 355.6 Nm rad^−1^, respectively (Dunn’s post hoc test, *p* < 0.0001, *n* = 126; figure 7*d*). The exoskeleton stiffness increased by approximately 5 times while the spring stiffness increased by more than six times.

During stance, the exoskeleton knee joint stiffness was higher than the phantom’s at each condition and this difference increased with increasing spring stiffness. For the 3.4 kN m^−1^ spring, the median phantom and exoskeleton stiffness values were 32.7 and 69.7 Nm rad^−1^, an approximately twofold increase. For the 21.7 kN m^−1^ spring, the phantom and exoskeleton stiffnesses were 51.6 and 355.6 Nm rad^−1^. For the stiffest spring, the phantom knee had a stiffness approximately seven times lower than that provided by the exoskeleton joint.

### 3.4. Power distribution

**Figure 8.**
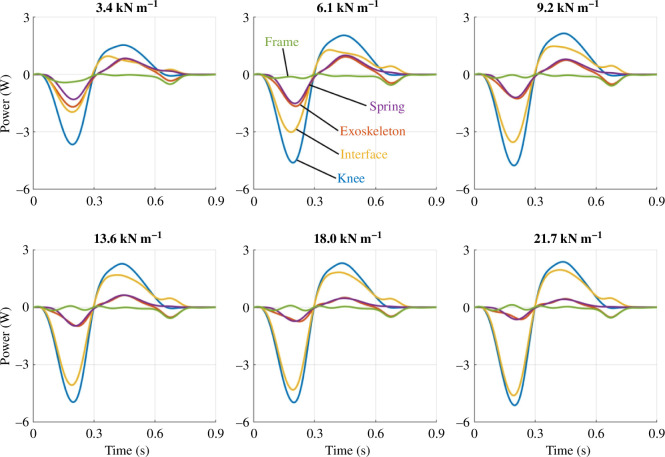
Power distributions across springs conditions. For each spring stiffness, the subplots show the mean power distribution of the knee, exoskeleton, physical interface, spring and exoskeleton frame during exoskeleton activation from early to terminal stance (0–45% of the gait cycle).

We partitioned the phantom/exoskeleton assembly into separate components to quantify the power distribution within the system and identify areas of energy loss, absorption and return. As shown in figure 8, the total knee power of the phantom/exoskeleton assembly (blue) contained power contributions of the exoskeleton (red) and physical interface (yellow). In turn, the exoskeleton (red) contained power contributions of its frame (green) and spring (purple). The interface power captured the power contained within the ballistics gel and exoskeleton straps. Similarly, the frame power captured the power within the exoskeleton bars, joints, dog clutch and chain transmission.

**Figure 9.**
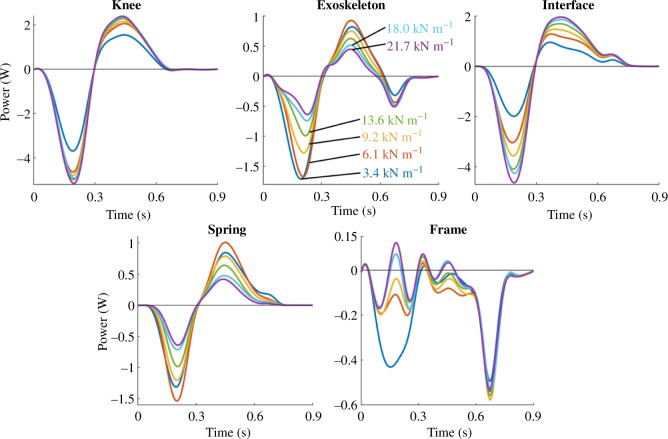
Power distributions across components. For each phantom/exoskeleton component, the subplots show the mean distribution of power across all spring conditions during exoskeleton activation from early to terminal stance (0–45% of the gait cycle). The trend indicates that exoskeleton power decreased with increasing stiffness while interface power increased. The resulting total knee power thus only shows small changes in power across springs.

As spring stiffness increased, the physical interface contributed more power to the total knee power relative to the exoskeleton contribution. Figure 8 shows the mean power of the knee (blue), exoskeleton (red), physical interface (yellow), frame (green) and spring (purple) for every spring condition during exoskeleton engagement. Alternatively, figure 9 presents the same power plots across individual components. The knee, exoskeleton, interface and spring powers underwent phases of energy absorption and return during the stance phase. However, the negative and positive total knee power peaks did not change considerably at the relatively high spring stiffnesses. Additionally, the positive and negative interface powers increased with increasing spring stiffness. The exoskeleton power contributions decreased simultaneously. Within the exoskeleton, the spring power decreased with increasing spring stiffness while the frame mostly dissipated power.

### 3.5. Work

**Figure 10.**
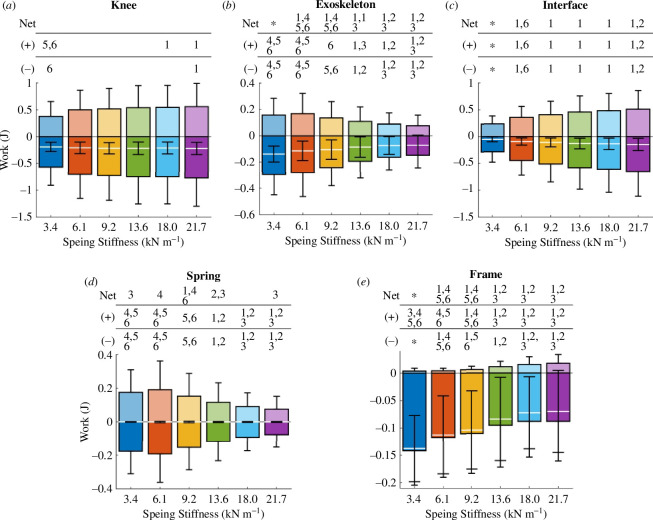
Exoskeleton and phantom work contributions. Bar graphs show the mean positive, negative and net work done during exoskeleton engagement by the phantom knee (*a*), exoskeleton (*b*), physical interface (*c*), spring (*d*), and exoskeleton frame (*e*) for a range spring stiffnesses. The net work is shown as a white line. Error bars denote standard deviations. (1), (2), (3), (4), (5), (6) = significantly different from springs 3.4, 6.1, 9.2, 13.6, 18.0, 21.7 kN m^−1^, respectively. (*) = significantly different from all other groups. The *p*-values for all the statistical tests are specified in electronic supplementary material, tables S4–S8.

**Figure 11.**
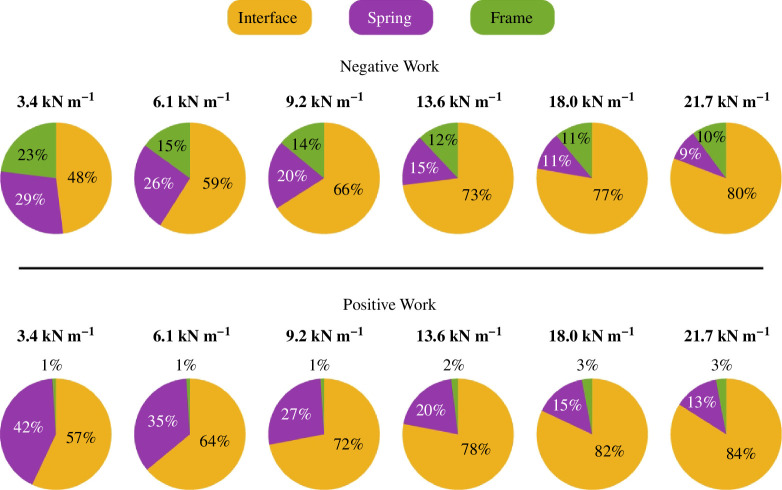
Relative work contributions. Pie charts show the relative contributions of the interface, spring and framework to the total negative and positive knee work. Interface work increased positive and negative work contributions relative to the other components as spring stiffness increased.

We integrated the power across the first 0.9 s of each stride of the phantom/exoskeleton assembly components to calculate their negative, positive and net work across all spring conditions. In figure 10, the mean positive and negative work of each component appear as bar plots in the positive and negative axes, respectively. Additionally, the mean net work is shown as a white line. Figure 11 shows the positive and negative work contributions of the interface, spring and frame relative to the knee work.

The negative, positive and net knee work differed only slightly across conditions (figure 10*a*). For the 3.4 and 21.7 kN m^−1^ springs, the median net work changed minimally from −0.18 to −0.20 J. For the same springs, the median positive work increased by 40% from 0.35 to 0.49 J. Similarly, the median negative work increased by 30% from −0.50 to −0.65 J.

The exoskeleton work decreased with increasing spring stiffness. The net, positive and negative work values were only significantly different across the spring stiffness extremes (figure 10*b*). For the 3.4 and 21.7 kN m^−1^ springs, the median exoskeleton net work decreased by approximately 70% from −0.13 to −0.04 J. Similarly, the median positive and negative work decreased by 60 and approximately 50%, from 0.13 to 0.05 J and from −0.27 to −0.12 J, respectively.

As spring stiffness went up, the physical interface not only absorbed and returned more energy, but also dissipated more energy. The net, positive and negative interface work were only significantly different across the extreme spring conditions (figure 10*c*). From the 3.4 to the 21.7 kN m^−1^ spring, the physical interface dissipated more than three times the net energy, from −0.04 to −0.14 J. Similarly, both the positive and negative median interface work increased from 0.22 to 0.44 J and from −0.26 to −0.59 J, respectively.

Although the springs absorbed and returned energy with negligible energy dissipation, their work contributions to the total knee energy decreased as spring stiffness increased. Across the spring conditions, the median net spring work did not exceed −7 $\times 10^{-4}$ J. From the 3.4 to the 21.7 kN m^−1^ spring conditions, the median positive work decreased by 60% from 0.15 to 0.06 J. Similarly, the median negative work decreased from −0.15 to −0.06 J.

The exoskeleton frame mostly dissipated energy, and its negative work contributions decreased with increasing spring stiffness. The median net framework decreased from −0.13 to −0.04 J as the stiffness increased from 3.4 to 21.7 kN m^−1^. For the same spring stiffness conditions, the median positive work values were negligible at 3 $\times 10^{-3}$ and 0.02 J, respectively. Additionally, the median negative frame work decreased from −0.14 to −0.06 J.

## 4. Discussion

We have described a methodology for testing and quantifying knee exoskeleton performance and energy losses using a synthetic lower limb phantom. The lower limb phantom emulated crouch gait kinematics and human soft tissue deformation. Our experimental results suggest that compliance of the physical human–exoskeleton interface drastically decreased exoskeleton assistance relative to the theoretical assistance. This assistance reduction was exacerbated by increasing exoskeleton spring stiffness. The results lay the framework for quantifying the mechanical energy distribution within the human–exoskeleton system to provide researchers with insights into improving knee exoskeleton design and iteration.

Increasing exoskeleton strength does not necessarily increase power transfer to the targeted joint. In our case, we had more than a sixfold increase in spring stiffness while keeping the phantom kinematics identical across conditions. Soft tissue compression decreased the exoskeleton’s range of motion about the knee joint. Like human soft tissue, the gel can compress under external loads and act as an in-series damped spring between the exoskeleton and the phantom knee. Under our testing scenario, a higher exoskeleton stiffness results in higher ballistic gel displacement and a lower exoskeleton range of motion. Shamaei *et al*. studied the angular excursion of a human knee and a passive knee exoskeleton over a range of exoskeleton spring stiffnesses [22]. They found that the exoskeleton excursion decreased with increasing spring stiffness while the human knee excursion remained relatively constant. Our findings add to the literature by demonstrating a method to track energy losses and transfer before reaching human testing of new exoskeleton technology. Few studies have assessed the effects of soft-tissue deformation on exoskeleton kinematics and energetics. Cherry *et al*. studied a knee exoskeleton for running and found that soft-tissue deformation of the leg probably decreased energy output from the exoskeleton by 50% [23,24]. In contrast, Lerner *et al*. found no significant mismatch between their cerebral palsy exoskeleton knee angle and the biological knee angle, but they had less than half the maximum exoskeleton joint torque in our study [25]. Our findings show that the difference in joint angle between exoskeleton and user skeleton (i.e. phantom skeleton in our case) will vary with exoskeleton torque. Using a soft tissue mechanical phantom provides researchers with a method for quantifying human–exoskeleton angle mismatches during device iteration and before human testing.

Our kinetics results showed that peak exoskeleton moments did not increase proportionally to spring stiffness. On the extreme ends, a more than sixfold increase in spring stiffness only increased the peak moments experienced by the phantom knee by approximately 50%. According to equation (2.1), the theoretical moment provided by the exoskeleton was expected to be approximately proportional to its tension spring stiffness. Exoskeleton simulations that fail to account for soft tissue deformation at the exoskeleton interface may misguide researchers on the actual level of assistance provided to its wearer, potentially hindering product development [26,27]. Besides kinetics, we show that soft tissue deformation affected the knee moment-angle plots of the phantom and exoskeleton. Similarly to the peak moments, a more than sixfold increase in spring stiffness only increased the phantom stiffness by approximately 60%. However, the same spring stiffness change increased exoskeleton stiffness approximately fivefold. Unlike exoskeleton stiffness, the phantom stiffness captured soft tissue compliance and hysteresis due to damping in the gel. Similar to the exoskeleton moments, equation (2.2) failed to capture the nonlinear behaviour of the phantom and exoskeleton stiffness with respect to the spring stiffness. The results again show that actual exoskeleton assistance can differ greatly from the theoretical one.

The quantification of energy distribution within the phantom–exoskeleton assembly explained the exoskeleton’s underperformance. The power distribution plots showed that the exoskeleton power decreased while the interface power increased with increasing spring stiffness. These observations support the idea that compliance at the exoskeleton interface decreased peak exoskeleton moments and phantom stiffness. Additionally, the decrease of spring power with increasing spring stiffness suggests that spring displacement decreased concurrently, supporting the results of a lower exoskeleton range of motion with higher spring stiffnesses. The decreasing exoskeleton work and concurrent increasing interface work resulted in small changes to the total work experienced by the phantom knee across spring conditions. A more than sixfold increase in spring stiffness only increased the negative and positive phantom knee work by 30 and 40%, respectively. Of the phantom–exoskeleton components, all but the frame absorbed and returned substantial mechanical energy. Unlike the spring and physical interface, the frame did not contain compliant materials that considerably deformed under load, thus acting as a main source of energy dissipation. Interestingly, the frame produced less negative work as spring stiffness increased. This result was probably caused by the concurrent decrease in exoskeleton work output due to the phantom–exoskeleton interface compliance. The power plots corroborate this idea, as the softer spring also had the highest negative power peak during the early stance. This peak occurred concurrently with the rise in exoskeleton joint angle, which was also the highest for the softest spring. We suspect that a higher exoskeleton transmission angular excursion and acceleration led to higher energy dissipation. The energy contributions of the human–exoskeleton components and interface have been understudied, given their importance in exoskeleton performance. Yandell *et al*. studied the power transmission and energy contributions of a soft ankle exosuit on a human [28]. Similar to our results, they found that the physical human–exosuit interface absorbed energy during exosuit loading and returned a portion of the energy during exosuit unloading. To our knowledge, no previous study has looked at the energetics of a knee exoskeleton considering the power contributions of the physical interface, exoskeleton frame and power source (spring for this exoskeleton). Our methodology provides researchers with a framework for estimating the energy distribution of robotic knee exoskeletons and optimizing the design process of robotic knee exoskeletons.

While not the main objective, we have also introduced a novel passive elastic knee exoskeleton. The exoskeleton provided knee extensor moments proportional to the change in knee angle during stance. Furthermore, the mechanical nature of the device makes it easy to use and lightweight at approximately 1.1 kg per limb. For comparison, active knee exoskeletons assisting crouch gait can weigh approximately 2.2 kg per limb [29]. The lightweight 3D-printed materials used to construct most of the exoskeleton frame and straps make the device easily adjustable to fit a wide range of user morphologies. The moments generated by the exoskeleton are also comparable to those of devices that aid walking in children with crouch gait. Our exoskeleton generated median peak moments during stance from 5.5 to 8.0 Nm, depending on the spring stiffness. Kennard *et al*. developed a passive knee exoskeleton using a bicycle braking rotor activated by a shoe pushrod to assist the knee during crouch gait [30]. Applying a 12 and 47 kg mass to the shoe pushrod, their device provided a dynamic braking torque within 1 and 5 Nm, respectively. A participant with crouch gait walking with their device experienced decreased hip flexion during gait. The active crouch gait exoskeleton developed by Lerner *et al*. provided subjects with a mean extensor knee moment of 0.17 and 0.06 Nm kg^−1^ during stance and swing, respectively [29]. For reference, 0.17 Nm kg^−1^ would translate to 5 Nm for a child the size of the phantom limb we used. Their exoskeleton improved the posture of children with crouch gait equivalent to the outcome of invasive orthopaedic surgery. Even though the physical interface limited our exoskeleton output, our device provided peak knee moments comparable to existing devices, and future work will include assessing the effects of device use during crouch gait.

Several study limitations must be addressed. First, the specific results of this study only apply to the specific exoskeleton and phantom limb construction. An exoskeleton with a different interface construction will probably present differing interface dynamics. Likewise, a phantom built with a different body mass index or ballistic gel density will probably show different interface dynamics. However, we expect to see similar trends among different exoskeleton designs targeting other joints across different subject morphologies. Another set of limitations is the simplified configuration of the phantom limb. The moulding process of the ballistic gel meant that its mechanical properties remained constant throughout the soft interface of the phantom. We used a 15% (w/v) ballistic gel density. The ballistic gel had a 20℃ temperature throughout the experiment, which corresponded to a 260 N m^−1^ dynamic stiffness measured with a MyotonPro (Myoton, Tallinn, Estonia) [10]. This dynamic stiffness fell within the range of biological dynamic stiffnesses of the lower limb muscles [7,22]. However, the biological soft-tissue dynamic stiffness can differ based on the muscle measured, muscle activity, leg measured, sex and age [2]. Another simplification is that the phantom knee simplifies the biological knee as a unicentric knee. The human knee is a complex joint with multiple degrees of freedom and shifting axes of rotation that are not captured by the current mechanical phantom, potentially leading to different results compared to a biological limb [31,32]. However, our exoskeleton acted during the stance phase of gait, where knee motion is relatively small (approx. median of 10°). While the phantom may differ from a real human leg, we expect that it is a close enough approximation of the human limb to provide us with information on the trends that soft-tissue deformation has on exoskeleton performance and energetics. Besides limb simplifications, we assumed that the exoskeleton did not change baseline crouch gait knee kinematics, which is appropriate based on examples in the literature [29,30]. Lastly, although the hexapod was limited to a 2 s stride time, we do not expect walking speed differences to drastically affect our results. The mean walking speed of the subject cohort used to model the phantom kinematics was 0.93 m s^−1^ [18]. Assuming an approximate 0.4 m step length, the kinematics of the phantom knee would correspond to a 0.4 m s^−1^ walking speed. The median peak difference between the exoskeleton and phantom knee angles during the stance phase at the stiffest spring was 7.9°. Using a back-of-the-envelope calculation and assuming that half of this difference in angular excursion was distributed equally between the thigh and shank, the displacement of the ballistics gel 15 cm away from the knee joint at a 3.9° rotational excursion would be approximately 1 cm. A 1 cm displacement over the 12 cm cross-sectional diameter of the thigh 15 cm away from the knee would roughly correspond to a 0.08 strain. This strain over the 0.3 s exoskeleton loading period would equal a strain rate of 0.3 s^−1^. The strain rate would approximately be 0.6 s^−1^ for a walking speed of 0.93 m s^−1^, assuming a one-to-ratio between walking speed and tissue strain rate. While the mechanical properties of ballistic gel and human soft tissue depend on strain rate [33–36], these differences are often seen across orders of magnitude in strain rate differences and at relatively higher strain rates.

We have demonstrated that soft tissue deformation at the exoskeleton interface may lead to unexpected results. In particular, our findings highlight that making the exoskeleton stronger (i.e. increasing spring stiffness in this case) does not substantially alter power transfer to the skeleton of the user (i.e. phantom limb in this case), despite similar joint kinematics, because soft tissue compresses more under high forces. Knee exoskeletons interfacing with the lower limb are especially susceptible to soft tissue effects due to the high soft tissue volumes at the thigh and shank. Using a lower limb phantom and a passive knee exoskeleton, we found that a stiffer spring led to decreased exoskeleton work output and increased physical interface work. The results suggest that a stiffer spring is not necessarily better at assisting the joint. While peak knee moments increased with spring stiffness, this increase was not proportional to the change in spring stiffness and excessive compression of human soft tissue caused by a stiffer spring may lead to tissue irritation and user rejection of the device [37,38]. Similarly, a more powerful motor is not necessarily better, as the increased power output from the device may instead be absorbed and dissipated by the physical interface. While an active exoskeleton may directly command torque, soft-tissue compliance may lead to a mismatch between the excursion of the exoskeleton and human joints, decreasing energy input to the targeted joint. We have introduced a methodology for estimating the performance and energy contributions of knee exoskeletons that will aid researchers in making informed design decisions while iterating and prototyping devices before human subject testing, ultimately improving the design process and testing of knee exoskeletons. It is valuable to note that the same approach could be used for hip, ankle, shoulder, elbow and wrist exoskeletons as well. It would require different mechanical phantoms but it would be a way to quantify energy transfer and losses prior to human testing.

## Data Availability

Supplementary materials are available at [39].

## References

[1] Kang MJ, Yoo HH. 2017 In vivo viscoelastic properties of human thigh under compression estimated by experimental results obtained with pendulum test. Int. J. Precis. Eng. Manuf. **18**, 1253–1262. (10.1007/s12541-017-0147-8)

[2] Ramazanoğlu E, Usgu S, Yakut Y. 2020 Assessment of the mechanical characteristics of the lower extremity muscles with myotonometric measurements in healthy individuals. Physiother. Quart. **28**, 1–12. (10.5114/pq.2020.97458)

[3] Bregman DJJ, Rozumalski A, Koops D, de Groot V, Schwartz M, Harlaar J. 2009 A new method for evaluating ankle foot orthosis characteristics: BRUCE. Gait Posture **30**, 144–149. (10.1016/j.gaitpost.2009.05.012) 19520576

[4] Dežman M, Massardi S, Pinto-Fernandez D, Grosu V, Rodriguez-Guerrero C, Babič J, Torricelli D. 2023 A mechatronic leg replica to benchmark human–exoskeleton physical interactions. Bioinspir. Biomim. **18**, 036009. (10.1088/1748-3190/accda8) 37068491

[5] Bessler-Etten J, Schaake L, Prange-Lasonder GB, Buurke JH. 2022 Assessing effects of exoskeleton misalignment on knee joint load during swing using an instrumented leg simulator. J. Neuroeng. Rehabil. **19**, 13. (10.1186/s12984-022-00990-z) 35090501 PMC8800279

[6] Massardi S, Rodriguez-Cianca D, Cenciarini M, Costa DC, Font-Llagunes JM, Moreno JC, Lancini M, Torricelli D. 2023 Systematic evaluation of a knee exoskeleton misalignment compensation mechanism using a robotic dummy leg. In 2023 Int. Conf. on Rehabilitation Robotics (ICORR), pp. 1–6. Singapore: IEEE. (10.1109/ICORR58425.2023.10304761) 37941226

[7] Ito T, Ayusawa K, Yoshida E, Kobayashi H. 2018 Evaluation of active wearable assistive devices with human posture reproduction using a humanoid robot. Adv. Robot. **32**, 635–645. (10.1080/01691864.2018.1490200)

[8] Van Loocke M, Lyons CG, Simms CK. 2008 Viscoelastic properties of passive skeletal muscle in compression: stress-relaxation behaviour and constitutive modelling. J. Biomech. **41**, 1555–1566. (10.1016/j.jbiomech.2008.02.007) 18396290

[9] Geerligs M, Peters GWM, Ackermans PAJ, Oomens CWJ, Baaijens FPT. 2008 Linear viscoelastic behavior of subcutaneous adipose tissue. Biorheology **45**, 677–688. (10.3233/BIR-2008-0517) 19065014

[10] Barrutia WS, Bratt J, Ferris DP. 2023 A human lower limb mechanical phantom for the testing of knee exoskeletons. IEEE Trans. Neural Syst. Rehabil. Eng. **31**, 2497–2506. (10.1109/TNSRE.2023.3276424) 37186529 PMC10311455

[11] Shamaei K, Sawicki GS, Dollar AM. 2013 Estimation of quasi-stiffness of the human knee in the stance phase of walking. PLoS One **8**, e59993. (10.1371/journal.pone.0059993) 23533662 PMC3606171

[12] Gesta A, Achiche S, Mohebbi A. 2023 Design considerations for the development of lower limb pediatric exoskeletons: a literature review. IEEE Trans. Med. Robot. Bionics **5**, 768–779. (10.1109/TMRB.2023.3310040)

[13] Park BK, Reed MP. 2015 Parametric body shape model of standing children aged 3-11 years. Ergonomics **58**, 1714–1725. (10.1080/00140139.2015.1033480) 25933223

[14] Viceconti M, Clapworthy G, Van Sint Jan S. 2008 The virtual physiological human—a European initiative for in silico human modelling. J. Physiol. Sci. **58**, 441–446. (10.2170/physiolsci.RP009908) 18928640

[15] Yin L, Chen K, Guo L, Cheng L, Wang F, Yang L. 2015 Identifying the functional flexion-extension axis of the knee: an in-vivo kinematics study. PLoS One **10**, e0128877. (10.1371/journal.pone.0128877) 26039711 PMC4454551

[16] Juliano TF, Forster AM, Drzal PL, Weerasooriya T, Moy P, VanLandingham MR. 2006 Multiscale mechanical characterization of biomimetic physically associating gels. J. Mater. Res. **21**, 2084–2092. (10.1557/jmr.2006.0254)

[17] Akduman V, Sari Z, Aydoğdu O. 2022 Evaluation of the effect of Bobath therapy on spasticity in children with cerebral palsy using subjective and objective methods. Göbeklitepe Sağlık Bilimleri Dergisi **5**, 79–85. (10.55433/gsbd-130)

[18] Lerner ZF, Damiano DL, Bulea TC. 2016 Estimating the mechanical behavior of the knee joint during crouch gait: implications for real-time motor control of robotic knee orthoses. IEEE Trans. Neural Syst. Rehabil. Eng. **24**, 621–629. (10.1109/TNSRE.2016.2550860) 27101612 PMC4914409

[19] Steele KM, Seth A, Hicks JL, Schwartz MS, Delp SL. 2010 Muscle contributions to support and progression during single-limb stance in crouch gait. J. Biomech. **43**, 2099–2105. (10.1016/j.jbiomech.2010.04.003) 20493489 PMC2914221

[20] Steele KM, Seth A, Hicks JL, Schwartz MH, Delp SL. 2013 Muscle contributions to vertical and fore-aft accelerations are altered in subjects with crouch gait. Gait Posture **38**, 86–91. (10.1016/j.gaitpost.2012.10.019) 23200083 PMC3600387

[21] Tabard-Fougère A, Rutz D, Pouliot-Laforte A, De Coulon G, Newman CJ, Armand S, Wegrzyk J. 2022 Are clinical impairments related to kinematic gait variability in children and young adults with cerebral palsy? Front. Hum. Neurosci. **16**, 816088. (10.3389/fnhum.2022.816088) 35308609 PMC8926298

[22] Shamaei K, Cenciarini M, Adams AA, Gregorczyk KN, Schiffman JM, Dollar AM. 2015 Biomechanical effects of stiffness in parallel with the knee joint during walking. IEEE Trans. Biomed. Eng. **62**, 2389–2401. (10.1109/TBME.2015.2428636) 25955513

[23] Cherry MS, Choi DJ, Deng KJ, Kota S, Ferris DP. 2006 Design and fabrication of an elastic knee orthosis: preliminary results. In Volume 2: 30th Annual Mechanisms and Robotics Conf., Parts A and B, pp. 565–573. Philadelphia, PA: ASMEDC. (10.1115/DETC2006-99622)

[24] Cherry MS, Kota S, Ferris DP. 2009 An elastic exoskeleton for assisting human running. In Volume 7: 33rd Mechanisms and Robotics Conf., Parts A and B, pp. 727–738. San Diego, CA: ASMEDC. (10.1115/DETC2009-87355)

[25] Lerner ZF, Damiano DL, Park HS, Gravunder AJ, Bulea TC. 2017 A robotic exoskeleton for treatment of crouch gait in children with cerebral palsy: design and initial application. IEEE Trans. Neural Syst. Rehabil. Eng. **25**, 650–659. (10.1109/TNSRE.2016.2595501) 27479974 PMC7995637

[26] Ostraich B, Riemer R. 2022 Design of a multi-joint passive exoskeleton for vertical jumping using optimal control. IEEE Trans. Neural Syst. Rehabil. Eng. **30**, 2815–2823. (10.1109/TNSRE.2022.3209575) 36155480

[27] Zhou L, Chen W, Chen W, Bai S, Zhang J, Wang J. 2020 Design of a passive lower limb exoskeleton for walking assistance with gravity compensation. Mech. Mach. Theory **150**, 103840. (10.1016/j.mechmachtheory.2020.103840)

[28] Yandell MB, Quinlivan BT, Popov D, Walsh C, Zelik KE. 2017 Physical interface dynamics alter how robotic exosuits augment human movement: implications for optimizing wearable assistive devices. J. Neuroeng. Rehabil. **14**, 40. (10.1186/s12984-017-0247-9) 28521803 PMC5437613

[29] Lerner ZF, Damiano DL, Bulea TC. 2017 A lower-extremity exoskeleton improves knee extension in children with crouch gait from cerebral palsy. Sci. Transl. Med. **9**, eaam9145. (10.1126/scitranslmed.aam9145) 28835518 PMC9993999

[30] Kennard M, Kadone H, Shimizu Y, Suzuki K. 2022 Passive exoskeleton with gait-based knee joint support for individuals with cerebral palsy. Sensors **22**, 8935. (10.3390/s22228935) 36433532 PMC9699336

[31] Masouros SD, Bull AMJ, Amis AA. 2010 (I) Biomechanics of the knee joint. Orthop. Trauma **24**, 84–91. (10.1016/j.mporth.2010.03.005)

[32] Bertomeu JMB, Lois JMB, Guillem RB, Del Pozo ÁP, Lacuesta J, Mollà CG, Luna PV, Pastor JP. 2007 Development of a hinge compatible with the kinematics of the knee joint. Prosthetics Orthotics Int. **31**, 371–383. (10.1080/03093640601095842) 18050008

[33] Salisbury CP, Cronin DS. 2009 Mechanical properties of ballistic gelatin at high deformation rates. Exp. Mech. **49**, 829–840. (10.1007/s11340-008-9207-4)

[34] Kwon J, Subhash G. 2010 Compressive strain rate sensitivity of ballistic gelatin. J. Biomech. **43**, 420–425. (10.1016/j.jbiomech.2009.10.008) 19863960

[35] Ottenio M, Tran D, Ní Annaidh A, Gilchrist MD, Bruyère K. 2015 Strain rate and anisotropy effects on the tensile failure characteristics of human skin. J. Mech. Behav. Biomed. Mater. **41**, 241–250. (10.1016/j.jmbbm.2014.10.006) 25455608

[36] Comley K, Fleck N. 2012 The compressive response of porcine adipose tissue from low to high strain rate. Int. J. Impact Eng. **46**, 1–10. (10.1016/j.ijimpeng.2011.12.009)

[37] Armitage L, Turner S, Sreenivasa M. 2021 Human-device interface pressure measurement in prosthetic, orthotic and exoskeleton applications: a systematic review. Med. Eng. Phys. **97**, 56–69. (10.1016/j.medengphy.2021.09.008) 34756339

[38] Rathore A, Wilcox M, Morgado Ramirez DZ, Loureiro R, Carlson T. 2016 Quantifying the human-robot interaction forces between a lower limb exoskeleton and healthy users. In 2016 38th Annual Int. Conf. of the IEEE Engineering in Medicine and Biology Society (EMBC), pp. 586–589. Orlando, FL: IEEE. (10.1109/EMBC.2016.7590770) 28268398

[39] Barrutia WS, Yumiceva A, Thompson ML, Ferris DP. 2024 Supplementary material from: Soft tissue can absorb surprising amounts of energy during knee exoskeleton use. Figshare. (10.6084/m9.figshare.c.7547677)39626746

